# Big Data Analytics in Large Cohorts: Opportunities and Challenges for Research in Hepatology

**DOI:** 10.1055/a-2599-3728

**Published:** 2025-05-21

**Authors:** Helen Ye Rim Huang, Kai Markus Schneider, Carolin Schneider

**Affiliations:** 1 Department of Internal Medicine III, Gastroenterology, Metabolic Diseases and Intensive Care, University Hospital RWTH Aachen, Aachen, Germany; 2 Division of Translational Medicine and Human Genetics, The Perelman School of Medicine, University of Pennsylvania, Philadelphia, Pennsylvania; 3 Department of Medicine I, Department of Gastroenterology and Hepatology, Faculty of Medicine and University Hospital Carl Gustav Carus, TUD Dresden University of Technology, Dresden, Germany; 4 Center for Regenerative Therapies Dresden (CRTD), Technische Universität (TU) Dresden, Dresden, Germany; 5 Else Kroener Fresenius Center for Digital Health, Faculty of Medicine and University Hospital Carl Gustav Carus, TUD Dresden University of Technology, Dresden, Germany; 6 The Institute for Translational Medicine and Therapeutics, The Perelman School of Medicine, University of Pennsylvania, Philadelphia, Pennsylvania

**Keywords:** big data, liver disease, cohorts, omics, artificial intelligence

## Abstract

Advances in big data analytics, precision medicine, and artificial intelligence are transforming hepatology, offering new insights into disease mechanisms, risk stratification, and therapeutic interventions. In this review, we explore how the integration of genetic studies, multi-omics data, and large-scale population cohorts has reshaped our understanding of liver disease, using steatotic liver disease as a prototype for data-driven discoveries in hepatology. We highlight the role of artificial intelligence in identifying patient subgroups, optimizing treatment strategies, and uncovering novel therapeutic targets. Furthermore, we discuss the importance of collaborative networks, open data initiatives, and implementation science in translating these findings into clinical practice. Although data-driven precision medicine holds great promise, its impact depends on structured approaches that ensure real-world adoption.

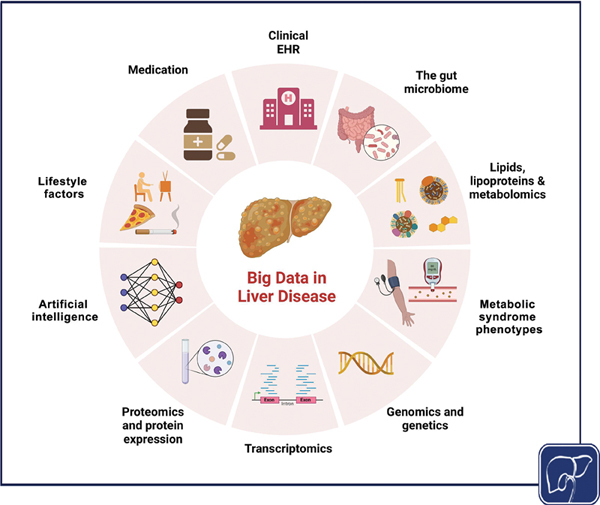

## Introduction

In our current era of digital medicine, public databases are an indispensable resource for understanding liver disease. Population-based databases are digital collections of data that are available to researchers from all over the world, 1 often after submitting an application and usually for a fee. They are a crucial tool in modern medical research, providing unparalleled access to information that is essential for hypothesis generation, testing, or validation. 2 These new sources that often also include increasingly multi-omic data are of interest in hepatology, as it has increased dimensionality and accessibility for researchers. The role that large databases can play in research is diverse, allowing researchers to perform various types of studies, including population-level comparative studies, disease monitoring and phenotyping, predictive modeling to improve prognosis or risk-stratification, or assessing unmet public health needs. 3 The integration of large-scale cohorts with increasingly sophisticated computational methods has transformed the landscape of liver disease research. As these technologies continue to evolve, their impact will only grow in the coming years. 4 By exploring the role of big data analytics in hepatology, we aim to highlight the opportunities it presents for both clinicians and researchers while addressing the challenges that must be overcome to fully unlock its potential.

**Fig. 1:**
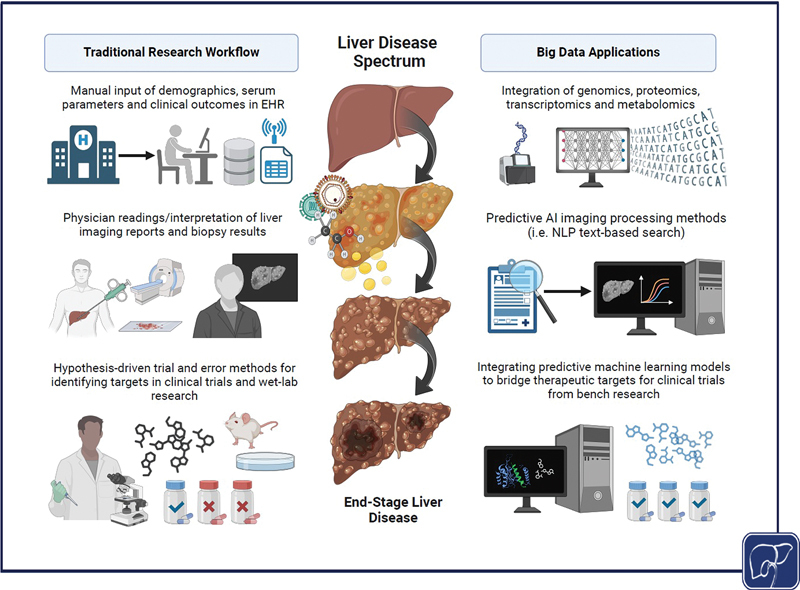
How big data can change the workflow of hepatology research.

Ongoing global scientific efforts aim to refine the classification of liver diseases and establish well-characterized phenotypic subgroups based on shared traits or treatment responses. Large-scale databases play a crucial role in this process, as they integrate diverse datasets: this includes patient demographics, lifestyle information including social history (i.e., alcohol intake, nutrition, pack years), genotypic information, detailed clinical data from collected specimens, results of imaging procedures (ultrasound, MRI, CT, or even FibroScan®), liver biopsy reports, microbiome data, and increasingly often omics data (lipidomics, metabolomics, proteomics, transcriptomics, etc.). A summary of these applications is depicted in Fig. 1 .

In population-based datasets, there are two different types of data: Unstructured and structured. 5 Structured data consists of quantitative metrics organized within predefined formats, such as electronic health records (EHRs), laboratory test results, and standardized clinical measurements. These datasets can be readily accessible through databases and are easier to analyze using conventional statistical and machine learning approaches.

However, unstructured data accounts for nearly 80% of all data and includes a wide range of complex, non-tabular information, such as physician notes, imaging reports, pathology slides, and genomic sequences. Unlike structured data, unstructured data are not directly searchable or analyzable within traditional data management systems. Extracting meaningful insights from these datasets requires advanced computational techniques, including natural language processing (NLP) for text mining 6 or deep learning for medical imaging to extract radiomic data. 7 By utilizing newer technologies with language models, 8 it is expected that unstructured data can become easily structured by creating more easily accessible summaries of medical information. A recent study demonstrated that large language models (like GPT4) are highly accurate for identifying cirrhosis and its complications from discharge summaries, outperforming traditional code-based classification, suggesting they could potentially augment or replace labor-intensive chart reviews in cirrhosis research cohort identification. 8 In hepatology, there is significant clinical utility in leveraging both structured and unstructured data due to the complexity of liver diseases and the need for comprehensive patient assessment. 9 Structured data, such as laboratory values (e.g., ALT, AST, bilirubin), imaging-derived biomarkers (e.g., liver stiffness from elastography), and standardized clinical scores (e.g., MELD, FIB-4), provide quantitative measures that facilitate disease classification, prognosis, and treatment decision-making. 10 Conversely, unstructured data, including physician notes, pathology reports, imaging scans, and endoscopic findings, contain valuable contextual information that structured data alone cannot capture. 11 Despite its clinical significance, unstructured data are often not systematically included in population-based datasets, limiting its potential for large-scale studies.

There are various types of population-based databases relevant to liver research, as summarized in Table 1 . Many of these databases have been collecting data over years or even decades, enabling longitudinal studies that provide valuable insights into disease progression and long-term outcomes.

**Table 1:** Some databases that may be relevant to liver research, with details of their respective data types

Region	Biobank	Number of participants	Type of data	Special features	Relevance for hepatology
**Europe**	KORA 73 (Cooperative Health Research in the Augsburg Region)	Over 18,000	Clinical, genetic, and environmental data	Focus on cardiovascular and environmental research	Data on liver disease and metabolic syndrome
	LURIC 74 (Ludwigshafen Risk and Cardiovascular Health Study)	Over 3,300	Genetic, metabolic, and inflammatory data	Focus on cardiovascular diseases	Information on interactions between cardiovascular and liver diseases
	NAKO 75 (German National Cohort)	Approximately 200,000	Clinical, genetic, and lifestyle data	One of the largest population-based studies in Germany	Comprehensive data on liver disease
	UK Biobank 76	Over 500,000	Genetic, clinical, and lifestyle data	Extensive genetic and medical data	Genetics, liver MRI, “omics”
	FinnGen 77	Over 500,000	Genetic and disease registry data	Combination of genetic and registry data	Genetics, “omics”
	Lifelines 78	Over 167,000	Clinical, questionnaire, biological data Microbiome	Longitudinal study on health and disease	Longitudinal data on the development of liver disease and microbiota
	Biobank Graz 79	Over 7.5 million samples	Biological samples, clinical data	One of the largest biobanks in Europe	Liver samples
	DeCODE Genetics 80	Over 160,000	Genetic and clinical data	Focus on the population of Iceland with extensive genealogical information	Genetic predispositions to liver diseases
	Our Future Health 81	Target of 5 million	Genetic, clinical, and lifestyle data	Aims to be the UK's largest health research program	Potential for large-scale studies on liver disease prevention and treatment
**United States**	Penn Medicine Biobank 82	60,000	Genetic and clinical data	Focus on personalized medicine and research	Data on genetic markers and personalized medicine in liver disease
	Mass General Brigham Biobank 83	Over 145,000	Genetic, clinical, and lifestyle data	Large-scale repository	Provides data for studies on liver disease risk factors and genetics
	Mayo Clinic Biobank 84	Over 21,000	Genetic, clinical, and lifestyle data	Comprehensive health data with a focus on individualized medicine	Resource for studies on genetic predispositions to liver diseases
	Kaiser Permanente Research Bank 85	Over 300,000	Genetic, clinical, and environmental data	Emphasis on health research across diverse populations	Enables studies on environmental and genetic factors in cancer
	All of Us 86	Target of 1 million	Genetic, environmental, and lifestyle data	Diversity and inclusion of a broad population	Diverse data on environmental and lifestyle factors that influence liver disease
	Million Veteran Program 87	Over 825,000	Genetic and military exposure data	Study of genetic and environmental factors in veterans	Effects of environmental and lifestyle factors on liver disease
	NHANES 88	Annually approximately 5,000	Clinical, nutritional, and health data	Longitudinal study on health and nutrition in the USA	Diet and lifestyle data, FibroScan
	MyCode Geisinger Health System 89	Over 200,000	Genetic and clinical data	System-wide biobank linked to electronic health records	Enables studies on genetic factors and personalized medicine approaches in liver disease
**Canada**	CARTaGENE 90	Approximately 40,000	Genetic, clinical, environmental, and lifestyle data	Population-based biobank in Quebec focusing on chronic diseases	Provides data for research on environmental, nutrition, and genetic risk factors for liver diseases
	Canadian Partnership for Tomorrow's Health (CanPath) 91	Over 300,000	Genetic, clinical, environmental, and lifestyle data	Pan-Canadian cohort integrating regional studies like CARTaGENE	Facilitates large-scale studies on liver disease risk factors across diverse Canadian populations
**Asia**	China Kadoorie Biobank 92	Approximately 500,000	Clinical and lifestyle data	Focus on chronic diseases in China	Data on common liver diseases in Asia and their risk factors
	BioBank Japan 93	Over 200,000	Genetic and clinical data	Focus on genetic data of Japanese individuals	Specific genetic data on liver disease in the Japanese population

To maximize the utility of these datasets, it is often necessary to systematically integrate data from multiple sources, such as linking omics data with cohort studies through a process known as data fusion. 12 This approach facilitates an integrative analysis of liver disease mechanisms, risk factors, and treatment responses across different populations and healthcare systems. A not yet existent uniform data structuring would allow for seamless integration and interpretation, making insights more actionable for both researchers and clinicians. 13 With well-organized and standardized datasets, big data holds great promise for improving clinical decision-making, improving diagnostic accuracy, enabling early risk prediction, and guiding personalized interventions for liver diseases.

## Opportunities in Big Data Analytics

**Fig. 2:**
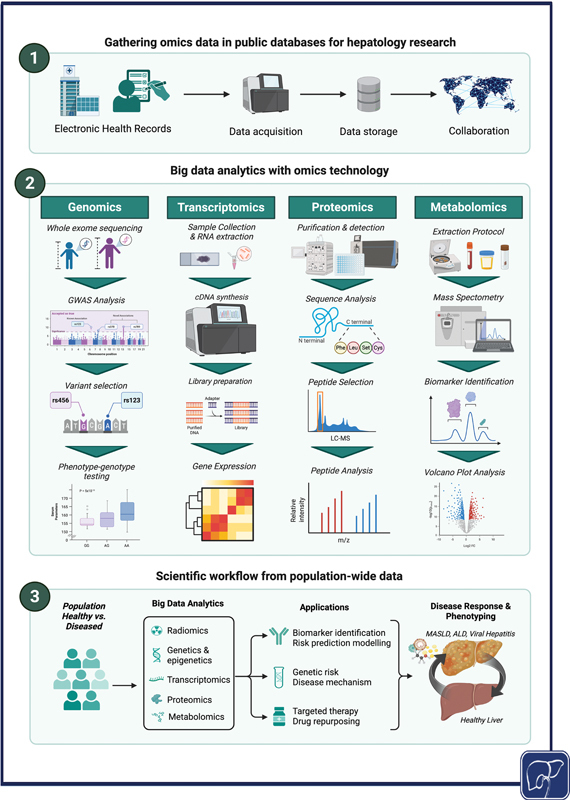
General schematic depicting examples of omics data and big data analytics.

In clinical hepatology, big data and omics based technology facilitates real-world evidence generation, enabling better stratification of patients and personalized medicine. 3 As disease entities in the field of hepatology are often heterogenous with a wide range of clinical phenotypes associated with age of onset, course of disease, and treatment responsiveness, analyzing big data can allow for refined subclassification of diseases through a concept called “phenomapping,” 14 allowing clinicians to better understand the pathogenesis of disease entities as well as develop predictive biomarkers/models. Fig. 2 summarizes how big data analytics could be applied to different forms of omics based data and its clinical applications described below.

### Biomarkers, Predictions, and Precision Therapy

Big data analytics enables the identification of biomarkers that can serve as diagnostic, prognostic, or therapeutic targets. The National Institute of Health identifies a biomarker as an objective measure and indicator of biologic function, pathophysiological processes, and response to treatment. 15 This has allowed for the application of big data in liver disease biomarker discovery. Moreover, we want to emphasize how novel targeted therapies for MASLD (e.g., TR-β agonists, GLP-1 receptor agonists, FGF21 analogs, and SGLT2 inhibitors) might leverage biomarker databases to facilitate personalized treatment approaches.

For identification of steatosis in the Kadoorie Biobank glutathione disulfide and diacylglycerol were found to be useful. 16 A study in UK Biobank has shown that lipidomic profiles, especially large HDL, may differentiate metabolic dysfunction-associated steatotic liver disease (MASLD) and combined metabolic dysfunction- and alcohol-related steatotic liver disease (MetALD). 17 As MASLD exhibits heterogeneity, a recent study identified two distinct phenotypic clusters identified through partitioning around medoids clustering: a liver-specific type with genetic links and rapid progression of chronic liver disease but limited cardiovascular risk, and a cardiometabolic type associated with dysglycemia, hypertriglyceridemia, and increased cardiovascular and diabetes risk. These clusters, validated across multiple cohorts, demonstrated distinct liver transcriptomic signatures. 18 Moreover, 32 biomarkers, including SHGB and ApoB, were associated with an increased incidence of HCC. 19 Plasma GDF15 was proposed to identify patients at risk of steatohepatitis. With increased accessibility in accessing population-based health datasets, clinicians and researchers can additionally check additional protein biomarkers associated with liver disease in the new proteome-phenome atlas from the UK Biobank. 20 In addition, biomarker discovery can lead to the validation of newer targeted therapies such as thyroid receptor β and FGF21 analogs.

Predictive models derived from big data are useful for stratifying patients based on their disease progression and treatment responses. Combining clinical parameters and sometimes omics data, these models can often predict outcomes such as fibrosis progression, HCC risk, or mortality with greater accuracy than traditional clinical scoring systems. Traditional liver injury markers such as transaminases as well as intrinsic liver function laboratories including albumin and coagulation panels have been critical in risk prediction of liver disease. 21 Traditional risk scores derived from large cohorts relevant in hepatology include the Model for End-stage Liver Disease (MELD) 22 score, FIB-4 Index, AST/ALT ratio, and the Albumin-to-Platelet Ratio Index (APRI). 23 However, the availability of big data has allowed researchers and clinicians to improve risk prediction with a combination of serum parameters and baseline demographics including more novel scores such as the LiverRisk score 24 and the Chronic Liver Disease (CLivD) score. 25 In the general population, it is difficult to diagnose liver disease as the presentation is generally asymptomatic. With the utilization of age, sex, alcohol intake, waist–hip ratio (as opposed to BMI), diabetes, smoking, and GGT, the CLivD score was developed to estimate 15-year risk of liver-related events.

Similarly in the study by Njei et al, the development of a machine learning model with FAST scores, liver stiffness measurements, and aminotransferase levels outperformed the ability to predict high-risk MASH compared with traditional FIB-4 or APRI scores. 26 For decompensation of cirrhosis the Early Prediction of Decompensation (EPOD) score was developed in three population-based studies. 27 Liver cancer, one of the most common cancers in the world, poses a serious public health burden due to high morbidity on diagnosis. Liu et al, using the UK Biobank cohort, developed a risk prediction model for liver cancer in a subset of over 300,000 participants without previous diagnoses of cancer on the basis of sociodemographic factors, physical measurements, lifestyle behaviors, and personal medical/family history. 28

One of the most crucial developments in hepatology research has come from genome-wide association studies (GWAS), uncovering the genetic basis of complex traits associated with elevated liver enzymes 29 or steatotic liver disease. 30 As shown in Table 1 many of the population-based studies include some type of genetic information, some even whole genome sequencing.

One prime example of how genetic has been used to advance hepatology is MASLD: Understanding genetic variants associated with MASLD has allowed us to better understand the functional mechanism leading to hepatic steatosis, as these genes are often involved in hepatic lipid metabolism, oxidative stress pathways, and inflammation leading to hepatic ballooning. 31 32 GWAS have promoted several genetic risk loci for MASLD by leveraging novel phenotypes including ALT proxies and magnetic resonance imaging proton density fractionated fat across various large cohorts. 30

As GWAS are insightful at drawing various associations with genetic variants in large-scale cohorts, a reverse methodology through phenome-wide association studies (PheWAS) and genotype-first approaches has been employed to map variant of interest within a single gene for multiple disease pathologies. In the setting of MASLD, this becomes relevant as its fundamental pathophysiological mechanisms arise from insulin resistance and dyslipidemia, thus leading to various phenotypic manifestations. Several genome-first approaches have been employed for the study of hepatic steatosis, all of which incorporate the data from large-scale biobanks with sometimes in vitro validation on cell models. 33 34 35 36 These advances highlight the importance of integrating genetic insights with clinical and multi-omics data to refine our understanding of liver disease pathophysiology. Although GWAS and PheWAS have significantly contributed to identifying risk loci and elucidating disease mechanisms, future studies will need to incorporate functional validation and mechanistic studies to translate these findings into clinical applications.

Notably, this genetic knowledge has already paved the way for the development of targeted therapies, exemplified by the first siRNA therapies for *PNPLA3* , 37 the most well-known genetic variant associated with MASLD. 31

## Advancing Precision Medicine and Drug Repurposing

Big data analytics facilitates the identification of patient subgroups that may benefit from targeted therapies. In hepatology, this approach is particularly relevant for tailoring treatments such as GLP-1 receptor agonists, immunotherapies, and antifibrotic agents to specific patient profiles. By refining treatment strategies, precision medicine can improve outcomes and reduce healthcare costs.

A key example of this progress is the rapid evolution of clinical trials leveraging big data, which has recently culminated in the first FDA-approved treatment for advanced fibrosis in MASH. 38 When starting this review in 2024, there were no FDA-approved treatment for MASH despite these cohorts being at a higher risk of histologically proved liver fibrosis. However, resmetirom, a liver-specific thyroid hormone receptor β-selective agonist, was shown to achieve MASH resolution in almost 30% of patients who received 100 mg of the medication as well as fibrosis improvement by at least one stage without worsening of the MASLD activity score. In addition, there is an emerging evidence that fibroblast growth factor 21 (FGF21) exhibits a substantial role in lipogenesis and hepatic insulin sensitivity, which has led to an additive effect to lipid profiles in combination with lifestyle changes such as diet. 39 As FGF21 also reflects liver fat accumulation, the exploration of transcriptomic data of MASLD models in mice suggested that upregulated FGF21 levels were protective against lipotoxicity and endoplasmic reticulum (ER) stress. As such, a phase 2b multicenter clinical trial on the FGF21 analog pegozafermin has been shown to improve fibrosis in biopsy-confirmed MASH. 40 New studies, which also include large cohorts, can now help pinpoint which patients might benefit most from these new drugs.

The increasing integration of precision medicine in hepatology extends beyond novel drug development to the potential repurposing of existing medications. 41 Given the metabolic constitution of MASLD, there is growing interest in leveraging anti-diabetic agents to improve liver-related outcomes. As insulin resistance and dyslipidemia coincide with the pathophysiology of hepatic steatosis, growing interest has focused on the role of anti-diabetic agents in improving clinical outcomes for these patients. With MASLD increasing in prevalence, institutions around the world have used large-scale population databases to investigate how glucagon-like peptide 1 receptor agonists (GLP-1 RAs) could improve liver inflammation. 42 Multiple large-scale studies have suggested that GLP-1 RAs could be repurposed to lower the risk of cirrhosis progression as well as reduce liver fat accumulation among patients with concurrent MASLD and type 2 diabetes. 43 44 Sodium glucose transporter 2 (SGLT2) inhibitors have also been shown to reduce histological steatosis as well as hepatic ballooning in MASLD likely by modulating inflammatory pathways associated with interleukins and AMP-activated protein kinase/mammalian target of rapamycin signaling. 45 46 Similarly, such mechanistic studies have allowed the study of SGLT2i in the context of hepatocellular carcinoma and diabetes, which showed improvements in lipid profiles and fibrosis. 47 However, there is not sufficient clinical evidence to suggest that SGLT2 inhibitors could be effectively repurposed. As for dyslipidemia and cardiovascular disease, studies in the UK Biobank as well as TriNetX cohort have suggested that statins, aspirin, and omega-3 intake could reduce the incidence of liver disease as well as mortality irrespective of genetic risk. 41 48 49

Although targeted personalized therapies and drug repurposing hold promise, further validation through well-powered clinical trials and mechanistic studies is essential. Moreover, there is a new player that is poised to further revolutionize precision hepatology.

## Applications of AI in Big Data Analytics

**Fig. 3:**
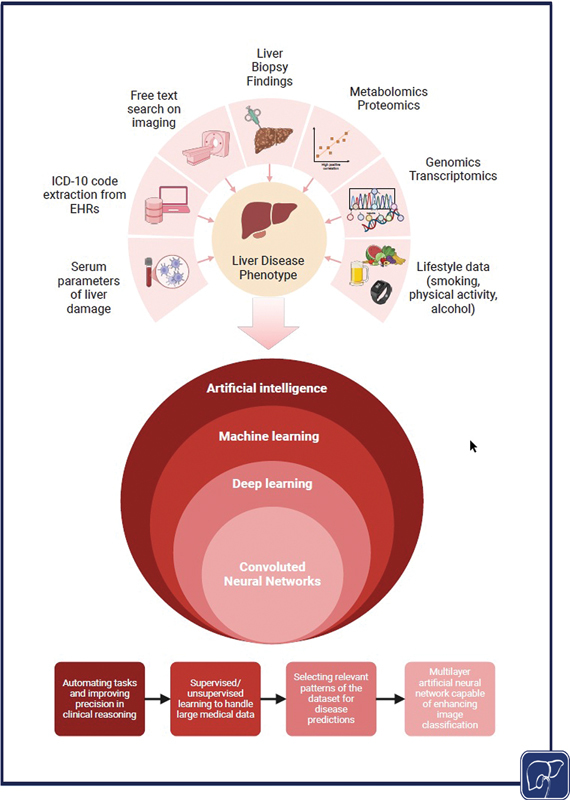
Applying artificial intelligence to big data in hepatology.

The application of artificial intelligence (AI) is revolutionizing liver research, 50 especially in the analysis of extensive data from databases. AI technologies, such as machine learning and deep learning, make it possible to detect complex patterns in large datasets that are too complex for human analysts. By developing predictive models, AI can improve the prediction of disease progression, treatment responses, and potential risk factors. 51 AI tools can automate time-consuming tasks, such as data processing and analysis, thus increasing research efficiency. 52 This is where the application of AI to population-based datasets is interesting, with Fig. 3 demonstrating a general schematic of different forms of data in hepatology that could benefit from AI processing.

Traditional statistical methods, such as regression models, have long served as foundational tools in hepatology research. Regression analysis remains a cornerstone for identifying associations between clinical variables, genetic risk factors, and disease outcomes, providing interpretable insights into MASLD progression and treatment response. Building on these statistical methods, machine learning techniques, including decision trees, support vector machines, and neural networks, offer more sophisticated ways to model complex interactions within large-scale datasets. 53 In a recent study by Yu et al, applications of deep learning algorithms such as spatio-temporal 3D convolution networks demonstrated robustness in accurately diagnosing HCC early based on triphasic CT liver scans. 54 Various methodologies stemming from AI models have allowed researchers to employ novel parameters such as anthropometric and body composition indices to accurately predict MASLD based on machine learning techniques applied to outcomes based on FibroScan classifications of steatosis and fibrosis. 55

A recent study identified how machine learning algorithms have been applied in hepatology: Random forest classifications followed by decision trees and support vector machines exhibited the highest average accuracy across 58 studies, with elevations in liver enzymes (i.e., aminotransferases) being the most utilized feature to define the liver phenotype. 56 Some examples of comparisons made between ML-based algorithms and conventional algorithms include early detection and diagnosis of reversible stages of steatotic liver disease, fibrosis risk in chronic hepatitis B, and development of hepatocellular carcinoma in metabolic liver disease. In a study done across patients with chronic viral hepatitis in Hong Kong, machine learning methods such as logistic regression, Adaboost, random forest classifiers, and decision trees were comparatively employed to construct accurate predictions of HCC in patients with chronic viral hepatitis based on already validated risk scores such as the HCC ridge score. 57

A comprehensive review paper by Ghosh et al well summarizes key areas that AI had been applied to study a wide range of liver diseases. 58 As AI continues to evolve, its integration into hepatology promises to refine risk stratification, enhance early diagnosis, and personalize treatment strategies for MASLD and related liver diseases. Although traditional regression models remain essential for hypothesis-driven research, advanced AI techniques not only offer the ability to uncover hidden patterns and optimize decision-making, but also pose some challenges.

## Challenges in Big Data for Hepatology

The increasing availability of diverse datasets and databases will revolutionize research in hepatology. Not only does it allow new hypotheses to be generated, but it also helps accelerate scientific discovery and innovation. However, challenges associated with the use of these databases—such as data quality, ethical concerns, privacy, and the need to distinguish between association and causality—must continue to be addressed with care and integrity.

### Data Quality and Heterogeneity

Although public databases offer a wealth of opportunities for liver research, there are also several pitfalls that researchers should be aware of. These challenges can influence the interpretation and use of the data and must be carefully considered to achieve valid and reliable research results. Big data are often plagued by issues such as missing data, variability in clinical measurements, and biases related to population selection. These challenges can lead to misleading conclusions if not addressed through robust data cleaning and normalization techniques. One of the biggest pitfalls is the quality and completeness of the data. In some cases, records may have gaps, be inconsistent, or contain errors. But also, the missing parameter in a cohort can be useful information; therefore, imputation is hard and can lead to erroneous conclusions. This can be especially the case if the data come from different sources or have been collected over long periods of time. A critical assessment of data quality is crucial to avoid erroneous conclusions. Another problem is the distortion of the data (bias). For example, databases could contain a disproportionate amount of data from certain demographic groups or regions, which can lead to a bias in research results. Moreover, loss of follow-up has to be accounted for. Researchers need to consider these potential biases and apply appropriate statistical methods to minimize them. 59 The results obtained from specific datasets may not be transferable to other populations or contexts. This is particularly relevant when research data come from a limited or special group of patients. Therefore, it is desirable to validate results in as many diverse datasets as possible. Correctly interpreting large and complex datasets also requires advanced statistical skills. Without proper methods and knowledge, researchers can draw misleading conclusions or miss important patterns and connections. It is also often underestimated that an advanced IT infrastructure is needed to carry out analyses according to the current *state of the art* .

### Computational and Technical Challenges

Despite promising research findings, the translation of big data insights into clinical practice remains challenging. Factors such as a lack of interoperability between systems, limited clinician training in data-driven tools, and resistance to change hinder the implementation of big data in routine hepatology care. Analyzing large datasets requires significant computational power. Scalability, efficient data storage, and the development of algorithms capable of handling high-dimensional data are critical technical hurdles in hepatology research. Similar to automated driving, the crucial question of responsibility and liability in the context of a decision based on the integration of AI algorithms in big data analytics is a point of contention. The challenges in hepatology are therefore also to enable a safe and evidence-based implementation of AI to support clinical decision-making. Various AI approaches have been used in hepatology, with a focus on predicting events, 60 histological analyses, 61 and the prediction of treatment response. 62 The inability of AI algorithms to take into account information from the direct interaction between patient and doctor is still an inherent limitation. AI algorithms will therefore not yet be able to replace direct interaction between doctor and patient. We see AI as a complementary tool to significantly improve patient–doctor interaction and patient care in general.

## Future Directions

### Collaborative Networks and Open Data

The concept of Open Science plays a central role in transforming hepatology research. 63 By disclosing research data and results, scientists worldwide can access and use the same resources to create high-quality and representative datasets. 64 It promotes the accessibility, transparency, and shareability of knowledge and data, which often applies to the population-based datasets. Moreover, medical collaborative networks aimed at sharing data sources from various different countries may provide a streamlined process to collaborate on these datasets. 65 Peng et al, in a bibliometric and social network analysis, suggested that at present, small academic groups are more popular in big data research with a broad range that makes interaction between different groups sparse. 66 Examples of collaborative initiatives that have aimed to accelerate the sharing of genomic and clinical data for the study of various disease processes include the Global Alliance for Genomics and Health (GA4GH) which aims to distribute access to central databases and allow researchers to reproduce their work by running established methods over the same underlying data. 67 In the broader scheme, other additional open sources including the European Health Data Evidence Network, the Observational Health Data Sciences and Informatics (ODHSI), and All of Us have paved way for establishing international networks of researchers with central databases of observational nature and establishing more streamlined pipelines to analyze data. With the ODHSI data, researchers have been able to identify metabolic risk factors in the cardiometabolic spectrum that may be relevant to patients with fatty liver compared with alcohol-related fatty liver disease. 68 In the United States, the All of Us program maintains large-scale population information to investigate various liver phenotypes, including drug-induced liver injury linked to antibiotics. 69

### Hypotheses versus Big Data

The growing emphasis on data sharing and collaborative networks has not only expanded access to biomedical datasets but has also influenced how research is conducted. Traditionally, biomedical research has been dominated by hypothesis-driven approaches, where studies were designed to test predefined mechanisms based on prior knowledge. However, the advent of big data has shifted the paradigm toward data-driven discovery, allowing researchers to uncover previously unknown patterns and relationships in hepatology without a predefined hypothesis. This shift has sparked a debate: Should research prioritize hypothesis testing or allow data to generate new hypotheses?

Big data approaches now enable the development of complex computational models integrating multi-omics datasets. 70 These models facilitate network-based disease characterization, allowing for a systems biology approach to understanding liver diseases. Rather than relying solely on predefined mechanistic pathways, machine learning algorithms can reveal hidden biomarker signatures and molecular interactions that may not have been considered in traditional hypothesis-driven research. Despite the power of data-driven approaches, the debate remains unresolved—hypothesis-driven research ensures biological interpretability and mechanistic understanding, while data-driven methods offer unparalleled discovery potential. The future of hepatology will likely depend on integrating both approaches, where data-driven insights inform hypothesis generation, leading to targeted experimental validation and ultimately improved clinical translation.

### Focus on Implementation Science

Regardless of the approach, the ultimate goal remains the same: improving patient care by translating research insights into clinical practice. This is where implementation science plays a critical role. Although big data and AI have transformed our ability to generate knowledge, their true impact depends on how effectively new findings, risk models, and therapeutic strategies are integrated into healthcare systems. Implementation science provides the framework to bridge this gap, ensuring that advances in hepatology research lead to tangible improvements. Implementation science focuses on promoting uptake of research into routine healthcare across clinical and organizational contexts. By improving the adoption and implementation of strategies derived from large cohorts, strategies must focus on identifying gaps in knowledge and practice to tailor interventions for a specific target audience. One example in hepatology is the Veterans Administration's National Hepatitis C Elimination Program, which successfully increased treatment rates by strengthening strategies to facilitate the transition to direct-acting antivirals from Year 1 to Year 2 while fostering stakeholder collaboration. 71(p2) Another example is the EASL-Lancet Commission on Liver Disease in Europe, which has advocated for policy changes and public health interventions to address the growing burden of liver disease. 72 Through a combination of data-driven decision-making and implementation strategies, this initiative has helped shape guidelines for MASLD screening and prevention, emphasizing the role of healthcare infrastructure in bridging the gap between research and practice. But still implementation of results derived from large cohorts is not the standard. Implementation, therefore, serves as a crucial bridge, ensuring that advances in precision medicine, big data, and AI are integrated into real-world settings to improve patient outcomes.

## Conclusion

Usage of large population-based cohorts will only increase in the next years. However, the true impact of these advancements depends on their successful implementation in clinical practice. Collaborative networks, open data initiatives, replication studies, and implementation science are essential to bridging the gap between large-scale discovery and real-world application. Moving forward, the synergy between data-driven research, clinical translation, and healthcare policy will be critical in transforming hepatology and improving patient outcomes on a global scale.

## Main Concepts and Learning Points

**Table:** 

Major concept	Key learning points	Role of large datasets
**Big data and multi-omics in hepatology**	- Integration of genetic, multi-omic, and clinical data increases liver disease understanding. - Integration of structured and unstructured data still holds some challenges.	- Enable identification of disease patterns, risk factors, and biomarkers at a population level. - Facilitate longitudinal studies to track disease evolution and treatment responses.
**Artificial intelligence in liver disease research**	- AI enables the identification of patient subgroups and personalized risk stratification. - Machine learning models help optimize treatment strategies and may uncover new therapeutic targets.	- Provide high-dimensional data needed to train robust AI models. - Improve predictive accuracy by capturing complex interactions between genetic, environmental, and clinical factors.
**Translational research and implementation science**	- Bridging the gap between AI-driven discoveries and clinical applications is essential. - Structured frameworks are needed to ensure real-world adoption of precision medicine.	- Large datasets support validation of AI models across diverse patient populations. - Help assess generalizability and effectiveness of predictive models in real-world settings.
**Collaborative networks and open data initiatives**	- Data sharing accelerates innovation and reproducibility. - Cross-disciplinary collaboration increases the development of effective precision medicine approaches.	- Enable meta-analyses and validation studies across different cohorts. - Support the development of standardized protocols for data integration and analysis.

## Notes

Lay Summary New technologies like big data, artificial intelligence, and precision medicine are changing how we understand and treat liver diseases. This review looks at how large research studies and genetic data help scientists learn more about conditions like steatotic liver disease. Artificial intelligence can sort patients into groups, suggest better treatments, and even help find new drugs. Collaboration between researchers and open data sharing are important to bring these discoveries into real-world healthcare. Although these advances have great potential, they need careful planning to make sure they truly help patients.
